# Improved Localization of Coronary Stents Based on Image Enhancement

**Published:** 2008-09

**Authors:** A. Ouled Zaid, I. Hadded, W. Belhaj, A. Bouallegue, S. Abdessalem, R. Mechmeche

**Affiliations:** 1 *SysCom Laboratory, National Engeeneering School of Tunis, Tunis, Tunisia;*; 2 *Department of Intensive care and Interventional Cardiology, La Rabta Hospital, Tunis, Tunisia*

**Keywords:** coronary angiography, stent implantation, rigid-body registration, generalized hough transform

## Abstract

Stent thrombosis remains a life threatening complication of percutaneous coronary interventions. The angiographic result of stent implantation is a high predictive factor of stent thrombosis. Nevertheless accurate placement of stents is hindered by the fact that most stents are only slightly radiopaque and hence difficult to see in typical coronary X-ray images. In this work, we propose a simple image guidance approach, making it easier to achieve optimum and complete intracoronary stent deployment. The main idea is to enhance the visibility of stents using an iterative landmark-based registration. After frame averaging over the series of registered frames, the resultant stent image is post processed to increase the contrast visibility. Preliminary simulation results show that despite its low computational cost, our method significantly improves the visibility of stent edge struts.

## INTRODUCTION

Stent thrombosis remains one of life threatening complications of incompletely deployed stents with a gap between stent struts and vessel wall. Moreover, the low radiographic contrast that characterizes intracororonary stents in angiographic sequences obstructs the assessment of the stent implantation outcome. Thereby, increasing radiographic contrast by using thicker stents or radiopaque coatings may have harmful clinical effects (1). To circumvent this problem, few solutions have been carried out. The most commonly used technique is the coronary Intravascular Ultrasounds (IVUS). Despite its simplicity, IVUS is an invasive technique and is significantly expensive in daily practice is significantly expensive. Another alternative is to improve stent visibility in X-ray images through image processing techniques. In (2) an effective technique has been developed to remove background structures and reduce random noise in coronary angiographic sequences. The main idea is to decompose the cine X-ray sequence into a sum of moving layers.

Due to the layer decomposition procedure, this technique is time consuming: several minutes are required to perform a layer decomposition. To be clinically useful, the processing time should not exceed few seconds, and it may eventually prove necessary to sacrifice some accuracy for the sake of speed.

More recently StentBoost Philips Medical System (3) has been designed as an interventional tool that improves the visualization of intracoronary stents in X-ray angiography. The StendBoost technique is principally based on motion-compensated noise reduction by landmark-based registration of multiple images. To remove background noise, registered frame averaging is performed on simulated angiograms. Although simulations appear to be quite realistic, clinical images may have a different distribution of contrast and noise to that found in a set of simulated sequences. Also, this procedure is costly in terms of time needed for computation.

In this work we have investigated the extension of the aforementioned method by two main contributions: first, to reduce the superimpositions and potential asynchronism that characterize the processed X-ray sequence, quasi synchronous images are identified by retaining either the frames acquired at the tele-diastole or tele-systole phases. Registration is then performed on frames showing the same anatomical locations of the heart. Second, a simple conventional enhancement process is accomplished passing up the use of simulated images. Thereby, after frame averaging over the series of registered frames, the resultant stent image is post-processed to improve stent contrast visibility.

Experimental results have shown that our method is faster than StentBoost system while providing comparable visual results. We also specify that our algorithm is entirely automatic. We have used it successfully without modification, not even tuning for the registration of a whole variety of coronary X-ray sequences.

The paper is structured as follows. In section 2 we describe our image enhancement scheme. In section 3, basic experimental results are presented and discussed. Section 4 concludes our discussion.

## PROPOSED METHOD

**Figure 1:**
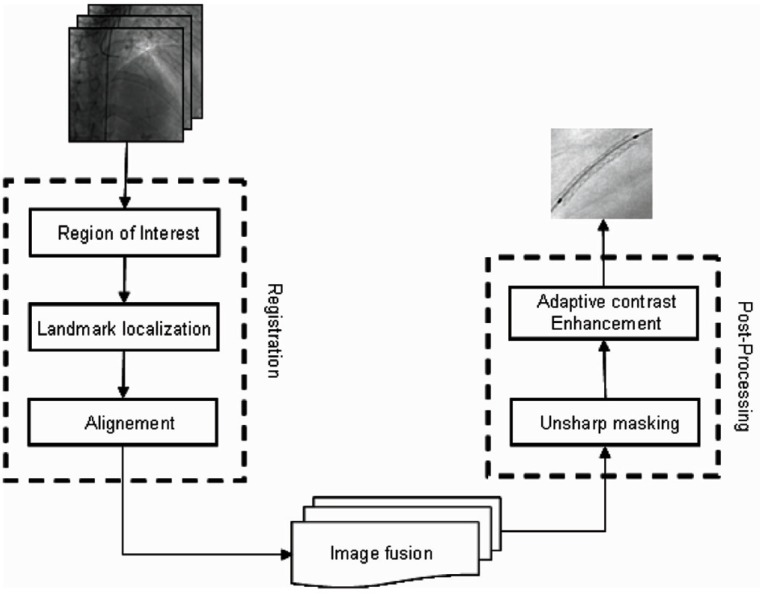
Flow chart of the proposed viewing scheme.

In order to improve image guidance of coronary stent deployment, we developed an algorithm which automatically enhances the intracoronary stent visibility from x-ray angiographic sequence. The key future of our enhancement scheme is principally based on motion-compensated noise reduction by landmark-based registration. Moreover, stent displacement is compensated by a registration process which is iteratively applied on frame pairs of the X-ray sequence. Image fusion (or frame averaging) over the series of registered frames is then carried out. Finally, in the third step, the resultant stent image is post-processed to improve the stent contrast visibility and remove background noise. The two last subsequent algorithmic parts are illustrated in a block diagram form as shown in Figure 1.

### Preprocessing

During the preprocessing step, three main prerequisites have been considered to reduce motion artifacts and increase the execution speed. First, the patients were asked to suspend their breathing during the image acquisition to consider only the cardiac motion. Second, rather than use all the primitives available in the set of 2D images in the sequence, we only treat the common stent zone. Finally, a pseudo-cardiac time for all sub-images in the sequence is identified. This information is computed from the image sequence information solely, without any external measures such as ECG signal. In our work, we retained quasi synchronous sub-images acquired at tele-diastole because it corresponds to the most relaxed and stable state along heart motion.

### Image fusion and landmark-based registration

The most common and easier registration technique is based on rigid-body transformation (4). The later can be used when shapes in the input image are unchanged, but the image is distorted by some combination of translation, rotation, and scaling. Since the stent can move, and rotate around a fixed axis, this spatial transformation type is well adapted to match two stent images from X-ray sequence. Typically, rigid-body transformations has four parameters, *T_x_*, *T_y_*, *s*, *θ*, which map a point (*x_1_, y_1_*) of the first image to a point (*x_2_, y_2_*) of the second image as follows:

(1) $$\left[\begin{array}{c} x_{2}\\ y_{2} \end{array}\right]=s\left[\begin{array}{cc} \cos\theta & -\sin\theta \\ \sin\theta & \cos\theta \end{array}\right]\left[\begin{array}{c} x_{1}\\ y_{2} \end{array}\right]+\left[\begin{array}{c} T_{x}\\ T_{y} \end{array}\right]$$

where *T_x_*, *T_y_* are the coordinates of translation vector, *s* is a scalar scale factor, and *R* is the rotation matrix. Because of the scalar scale factor *s*, the rigid-body transformation allows changes in length relative to the original image, but it is the same in both *x* and *y*. To solve the four unknown parameters, at least two control-points (or landmarks) are needed. Since the stent zone in coronary angiographic sequence, is supposed to undergo the same type of motion as the deflated balloons. Hence, it is possible to parameterize the rigid-body transformation by the balloon displacement which describes the global motion.

Contrary to the approach suggested by (3) that picks landmark pairs manually, we propose to automatically determine control points positions, depending on the a priori knowledge of their template shape, using the Extended Generalized Hough Transform (EGHT).

**Figure 2:**
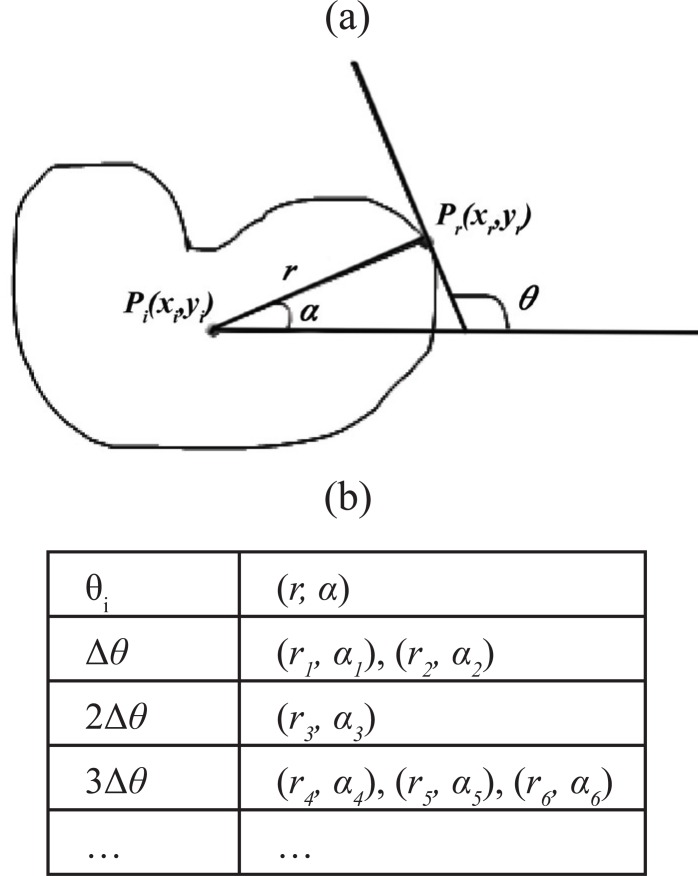
Generalized Hough Transform. (a), Illustration of the geometry for building the R-table; (b), The R-table format.

The Generalized Hough Transform (5) is a computationally efficient technique of detecting or locating shapes in binary images. This method uses a lookup table, termed the R-table, for an arbitrary shape, so no analytical description for the shape is necessary. Figure 2 illustrates the geometry for building the R-table and its format. Let *P_r_*(*x_r_, y_r_*) be a reference point which is the origin of an axis system fixed in the template shape. An arbitrary point on the template boundary *P_i_*(*x_i_, y_i_*) is specified by Equation 2 and Equation 3.

(2) $$r=\sqrt{\left(y_{i}^{2}-y_{r}^{2}\right)-\left(x_{i}^{2}-x_{r}^{2}\right)}$$

(3) $$\alpha =\tan^{-1}\left(\frac{y_{i}-y_{r}}{x_{i}-x_{r}}\right)$$

where *r* is the Euclidean distance from the reference point to the boundary point, and α is the angle of the line connecting *P_i_*(*x_i_, y_i_*) and *Pr*(*x_r_, y_r_*). The pairs of (*r, α*) are then indexed by local edge direction angle *θ_i_*, which is determined by the intersection of the tangent line through *P_i_*(*x_i_, y_i_*) and the horizontal axis. The format of the R-table is then defined in terms of discrete value *θ_i_* and corresponding (*γ_i_, α_i_*) pairs, as illustrated in Figure 2(b).

Although theory does not set constraints for the location of the reference point, a common practice is to choose *P_r_* to be inside the template. In our study, *P_r_* is positioned as the center of the minimum bounding box that encloses the template, which corresponds to the deflated balloon midpoint.

After registering features related to stent in the sub-images with respect to the image referential, pixels intensities means over the succeeding registered sub-images are determined to obtain the resultant stent image.

### Post-processing

Due to the mean fusion process, resultant coronary stent image is corrupted by additive blurring or smoothing. To improve the stent structure visibility we used a contrast enhancement scheme based on blurred filtering followed by histogram normalization.

## EXPERIMENTS AND RESULTS

### Visual image quality assessment

**Figure 3:**
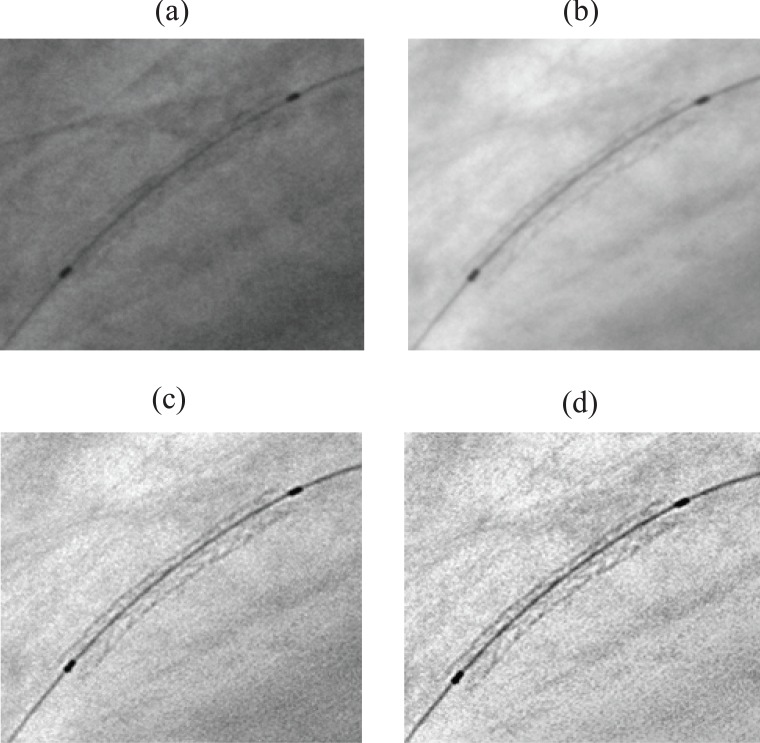
A cropped stent image. (a), original; (b), result after registration and fusion; (c), after blurred filtering; (d), after histogram normalization.

**Figure 4:**
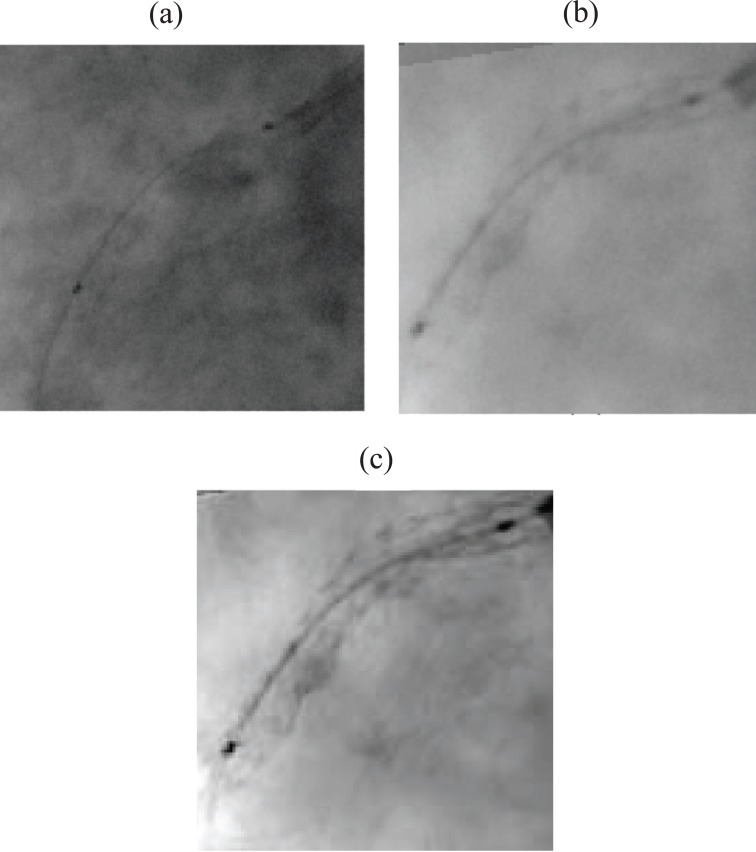
A bad quality X-ray image of coronary stent; (a), original image; (b), a crop region after StentBoost enhancement; (c), a crop region after our enhancement processing.

In this section, we present some preliminary validation results obtained on patient datasets of randomly selected coronary angiograms. Each coronary X-ray image is consists of 512 rows by 512 columns with 256 levels of gray, and stored in uncompressed DICOM format. Our validation tests are only based on visual image quality assessment. Due to the lack of availability of clinical material, we could not compare the quantitative stent measurements with other methods such as IVUS. However, we do plan to further validate our algorithm by comparing the quantitative stent measurements with IVUS results as soon as they are available. The results indicated that our technique was effective in enhancing stent visibility in most cases. Figure 3 shows a significant quality improvement in a test angiogram using the successive steps of our processing scheme. It appears clear that the visibility is significantly increased, stent contrast is greatly enhanced, and there is very little blurring. Our method has also been compared to StentBoost system. Figure 4 illustrates the enhancement result on a bad quality X-ray image of coronary stent, using StentBoost and our enhancement framework. From this figure, we can notice that our method performs better than StentBoost system.

**Figure 5:**
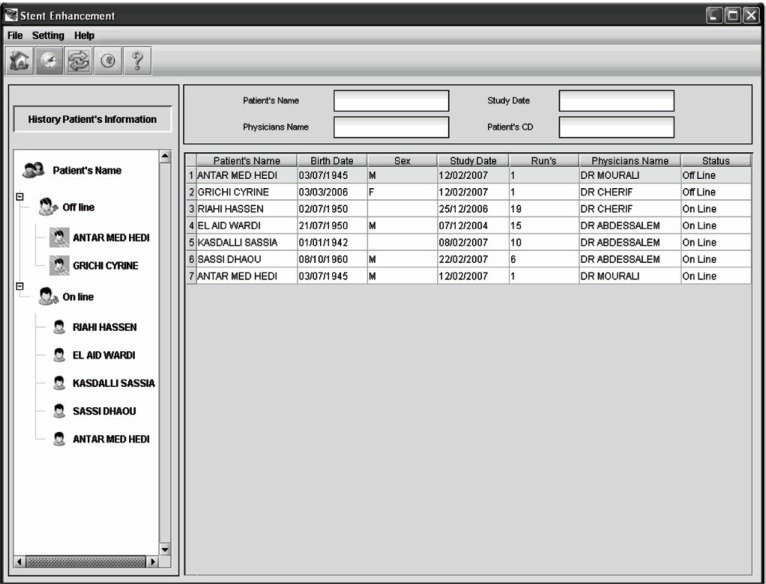
Screen shot of the main window in the viewer tool showing the patient’s historic guidance.

To make the use of our stent image enhancement application easier, we have integrated it on an archiving system with viewer tool. The latter is shown in Figure 5.

**Figure 6:**
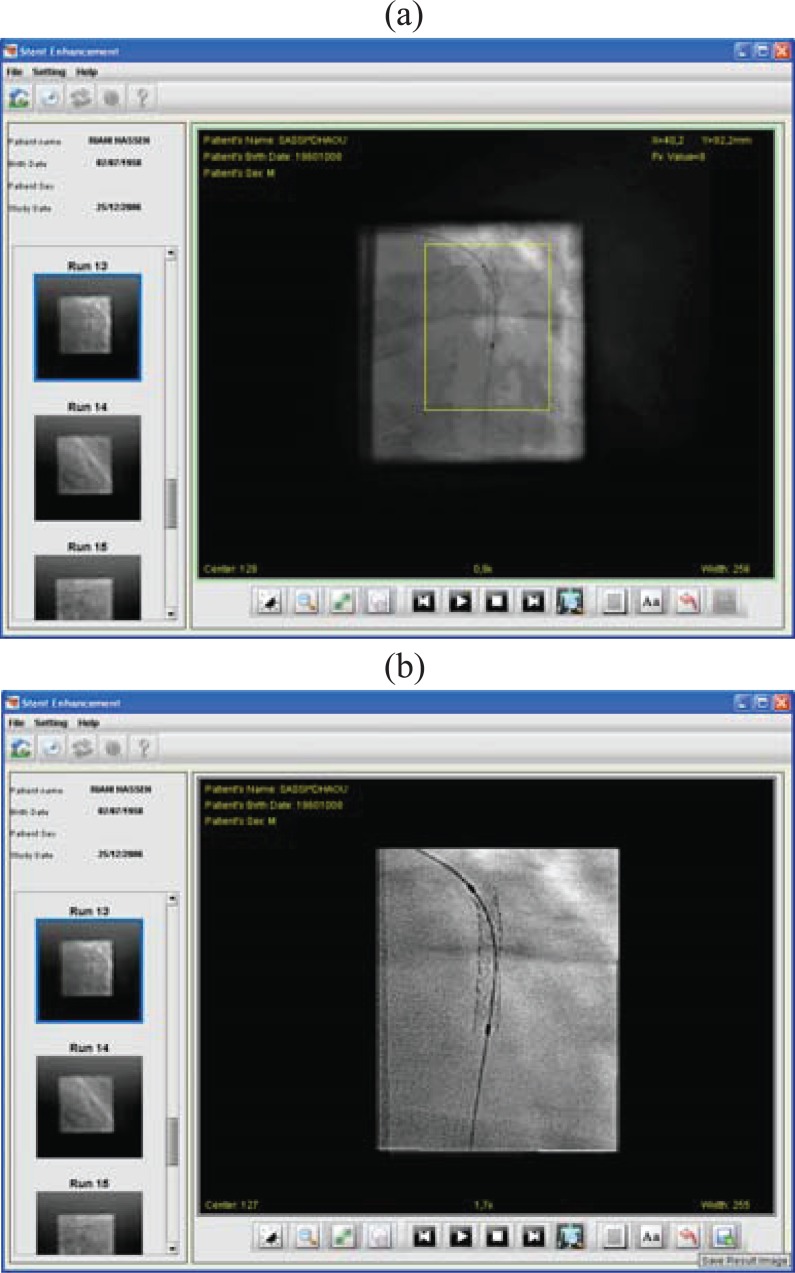
Screen shots of stent enhancement tab panel depicting. (a), a cropped stent image with a list of coronary angiogram sequences; (b), Resultant stent image after the enhancement.

It allows cardiologists to login into a database and enable collection and displaying of a given X-ray sequence from the database, depending on a chosen parameter such as patient identifier, cardiologist name or intervention date. In developing a medical viewer tool, it is essential to take into account the fact that cardiologists are providing this while caring for their patients during invasive interventional procedures. As demonstrated by Figure 6(a), the user can select one among multiple coronary angiographic sequences to be processed and displays the first 2D image from the collected run.

As illustrated in Figure 6(b), for each treated sequence, the user is shown the image, to be investigated, on a tabbed interface with the entire dynamic image on the main tab, and the corresponding resultant stent image (region of interest) on the other tab.

### Computation times

Another advantage of our stent enhancement framework is execution time. During the development of the algorithm, all procedures were implemented using JAVA cross-platform language on Intel Core Duo T2400 1.8 GHz computer with 1 Go of RAM. Although the registration preprocessing step needs to be carried out iteratively in each sub-image of the sequence, it is performed quickly. Table 1 shows the execution time of the proposed approach for different coronary X-ray sequences as a function of the number of frames. Based on Table 1, we can notice that the framework of our stent enhancement algorithm is computationally very effective. It only requires a mean of 8 seconds to process a run of about 60 frames, which is lower than Stentboost scheme that requires a processing time of about 2 minutes for a 60 frames run.

**Table 1:** Execution time for different tested angiograms

Sequence	Number of frames	Execution Time (seconds)

Sequence 1	51	6.992
Sequence 2	51	6.992
Sequence 3	38	4.250
Sequence 4	46	6.878
Sequence 5	49	6.093

## CONCLUSION

In this work, we proposed a visual system in order to reduce life threatening stent thrombosis. Our approach is principally based on a combination of simple image processing techniques that can noticeably improve the stent visibility and enable interventional cardiologists assess stent deployment more accurately while using the least amount of radiation. The proposed scheme has the distinct advantage that it contributes to an automation of the assessment tool during interventional procedures. This is one of the objectives aimed at, taking into account the considered request.
